# Correlates of HIV Testing Experience among Migrant Workers from Myanmar Residing in Thailand: A Secondary Data Analysis

**DOI:** 10.1371/journal.pone.0154669

**Published:** 2016-05-03

**Authors:** Patou Masika Musumari, Chalermpol Chamchan

**Affiliations:** 1 Department of Global Health and Socio-epidemiology, Kyoto University School of Public Health, Kyoto, Japan; 2 Institute for Population and Social Research, Mahidol University Salaya, Phutthamonthon, Nakhon Pathom, Thailand; Tulane University School of Public Health, UNITED STATES

## Abstract

**Background:**

Thailand continues to attract an increasing number of migrant workers (MW) from neighboring countries including mainly Myanmar, Cambodia, and Laos; however, little is known about the extent to which MWs from these countries have access to HIV prevention, treatment, and care services. We used data from the baseline survey of the Prevention of HIV/AIDS among MWs in Thailand (PHAMIT-2) project to document the prevalence of, and factors associated with, HIV testing among MWs from Myanmar, the largest group of MWs in Thailand.

**Methods and Findings:**

The baseline survey of PHAMIT-2 was conducted in 2010 among MWs from Myanmar, Cambodia, and Laos in 10 purposely-selected provinces of Thailand. Of the 1,034 participants who qualified for the analysis to identify correlates of HIV testing, only 5.3% reported ever having been tested for HIV. Factors associated with HIV testing included having a secondary or higher education level (AOR, 2.58; CI, 1.36–4.90; *P* = 0.004), being female (AOR, 1.96; CI, 1.05–3.66; *P* = 0.033), knowing someone who died of AIDS (AOR, 1.81; CI, 1.00–3.27; *P* = 0.048), working in the fishery sector (AOR, 2.51; CI, 1.28–4.92; *P* = 0.007), and not having a work permit (AOR, 3.71; CI, 1.36–10.13; *P* = 0.010).

**Conclusion:**

Our study, in addition to revealing significantly low HIV testing among MWs from Myanmar, identifies important barriers to HIV testing which could be addressed through interventions that promote migrants’ culturally-sensitive and friendly service, for example by facilitating flow of information about places for HIV testing, availability of language assistance, and ensuring confidentiality of HIV testing.

## Introduction

Labor migration is a global human phenomenon fueled by a wide array of factors such as globalization, demographic shifts, conflicts, income inequalities and climate change. The current statistics indicate that there are approximately 232 million international migrants [1, 2], and that more than 90% of international migration is related to seeking employment [3]. Thailand has achieved tremendous progress in its economic development. In less than a generation, the country has shifted away from a low-income status to become an upper-income country [4]. As such, Thailand has become a magnet country, attracting an increasing number of migrants from its less-developed neighboring countries, namely Myanmar, Cambodia, and Laos. There are approximately 3.6 million migrant workers (MWs), from Myanmar, Cambodia, and Laos, in Thailand, including both documented and undocumented migrants, with or without work permit [5]. Thailand, being a member of the AEC (ASEAN Economic Community), an organization that endeavors to promote economic integration in the region, including free movement of labor [6], is expected to experience a continued increase in the number of its migrant workers, with downstream impact on its public health system [7].

Thailand has a success story in reducing rates of HIV infection among the Thai population, which is overwhelmingly cited as an example of an effective HIV prevention program [8–10]. However, the country still holds one of the highest rates of adult HIV prevalence in Asia [11], and HIV infection has remained fairly high among high-risk individuals, including intravenous drug users (IDUs), men who have sex with men (MSM), and commercial sex workers [12]. In Thailand, MWs are recognized as a priority group for HIV prevention, treatment and care [12]. The vulnerability of MWs to HIV infection is widely established. Several studies in Asia have documented a number of risk factors among MWs including but not limited to low levels of AIDS knowledge, low perceived risk for HIV/STIs (sexually transmitted infection), high levels of risky sexual behaviors, and limited access to health care [13–20]. Similarly, many studies in Thailand showed that MWs engaged in behavioral patterns that increased the risk of HIV infection. Low rates of condom use [21–23] and high frequency of visits to sex workers [21, 22], compounded with poor access to health care services [24, 25], were commonly observed among migrant workers in Thailand.

Despite the documented profile of risky sexual behaviors among MWs in Thailand, there are still extensive knowledge gaps regarding HIV testing in this population. HIV testing is the gateway to effective HIV prevention and treatment. Many studies have shown that awareness of HIV status is associated with substantial reduction of risky sexual behaviors among HIV-infected individuals, and persons with HIV-negative status are less likely to engage in risky sexual behaviors after they become aware of their HIV status [26]. HIV testing fosters timely linkage to antiretroviral therapy (ART), which in turn is associated with positive clinical outcomes, and reduced risk of HIV transmission at both individual [27, 28] and population levels [29]. However, the proportion of individuals who know their HIV status remains significantly low. At the global level, only 50% of the population were aware of their HIV status as of the end of 2012 [30]. In Thailand, studies on HIV testing in Thailand, as a reflection of the concentrated nature of the epidemic in the country [12, 31], have mostly focused on populations most at risk (youth and adults) including MSM, female and male sex workers, and IDUs, with HIV testing rates ranging from 23–76% [32–36]. Little is known about HIV testing behavior among MWs in Thailand, including factors that facilitate and restrain the uptake of HIV testing services in this population. Such information is crucial for guiding interventions to increase HIV testing among MWs in Thailand, and ultimately, to prevent HIV infection and promote timely linkage to HIV care and treatment.

The current study uses data from the baseline survey of the Prevention of HIV/AIDS among MWs in Thailand (PHAMIT-2) project to document the prevalence of, and factors associated with, HIV testing among MWs from Myanmar residing in Thailand. PHAMIT-2 is a GFATM (Global Fund to Fight AIDS, Tuberculosis, and Malaria)-funded project designed to reduce new HIV infections among MWs mainly from Myanmar, Cambodia, and Laos [37]. The current analysis is restricted to MWs from Myanmar, which is the largest group of MWs in Thailand.

## Methods

### Ethics Statement

This study has received ethical approval from the Institutional Review Board (IRB) of the Institute For social and Population Research (IPSR) Mahidol University (IRB Number: IRB0001007). All the participants in the PHAMIT-2 project provided informed consent before being interviewed. Participants were first informed in their own language about the study’s objectives, their roles and rights to give or not to give any information during the interview, confidentiality of the personal data and way that findings are presented. They could provide either written or verbal informed consent. The verbal consent was mostly the case of those who could not write or were not comfortable to provide their written name or signature on the consent form. In such case, the interviewer would ask for permission to put his or her name/signature on the consent form on behalf of the respondent. This consent procedure was clarified to and approved by the IPSR-IRB.

### PHAMIT-2 project

PHAMIT-2 is one of the prongs of the GFATM-funded program called Comprehensive HIV Prevention among Most at risk populations by Promoting Integrated Outreach and Networking (CHAMPION). CHAMPION’s target populations include female sex workers (FSW), men who have sex with men (MSM), intravenous drug users (IDU), young people, and cross-border MWs. The implementing bodies were the Thai Ministry of Public health for prevention among FSW and MSM, Population Services International (PSI) Foundation for IDU, and the Raks Thai Foundation (RTF) for cross-border MWs under the PHAMIT-2 project. PHAMIT-2 included a baseline survey in 2010, a mid-term evaluation survey in 2012, and impact survey in 2013. The project was designed to ultimately reach 117 districts in 37 provinces, with a focus on 34 provinces during the first year of implementation. Of the 34 provinces, 22 majorly were home to MWs from Myanmar, 4 provinces mostly housed MWs from Cambodia, and MWs from Laos predominantly occupied 8 other provinces [37]. The current paper focuses on the baseline survey of the PHAMIT-2 project.

### Participants and Setting

The PHAMIT baseline survey was conducted from January through August 2010. In this survey, MWs were defined as males and females from Myanmar, Cambodia, and Laos, aged 18–49 years, who have resided in Thailand for at least 3 months, with or without a work permit, and are working in fisheries, seafood processing factories, construction or agriculture in 34 provinces covered by the PHAMIT project. Since there was not any census of all MWs in Thailand, and because of the unregistered status of many MWs, the survey team relied on the PHAMIT-2 partner agencies for the estimation of the number of MWs present in any given province. The baseline survey included 10 provinces that had the largest number of MWs among the 34 provinces covered by the PHAMIT-2 project. Of the 10 purposely-selected provinces, six were selected from the 22 provinces, which predominantly housed MWs from Myanmar, and 2 provinces were selected from each of the groups of provinces where the majority of MWs from Cambodia and Laos resided. A sample size of four hundred participants was assigned to each of the 10 provinces based on the Yamane’s Formula [38] and considerations regarding feasibility and the need to ensure representativeness of the entire population of migrants. Each province had 40 recruitment sites distributed in proportion to the estimated size of the entire population of MWs at the district and sub-district levels. Participants were recruited using a snowball sampling technique driven by chain-referral, the first respondent serving as the “seeder,” and referring the other “seeds” with a maximum of ten seeds for a given recruitment site. Overall, the baseline survey targeted 4,000 participants for the total sample size including 2,400 MWs from Myanmar.

### Survey Instrument

A structured questionnaire was developed by the survey team, and was reviewed by the PHAMIT-2 partner agencies. Prior to conducting the actual field work, the questionnaire was pre-tested, and refined to ensure clarity and fluidness of the administration. The questionnaire was administered by trained interviews in Thai, English, Burmese languages (Burmese and Mon) or Khmer, depending on the language the participants were most comfortable with. The questionnaire survey included items that addressed participants’ socio-economic and demographic characteristics, knowledge and behavioral questions that are consistent with the United Nations General Assembly Special Session (UNGASS) and GFATM indicators, and other relevant questions that fit the objectives of the PHAMIT-2 project. A full description of the PHAMIT-2 project, including details on items used in the questionnaire is provided elsewhere [37].

### Variables

We selected a set of variables to document the prevalence and correlates of HIV testing among MWs from Myanmar in Thailand. The primary outcome of this study was HIV testing experience, and was assessed using the item “have you ever been tested for HIV?” The covariates were selected based on the existing literature [13] and common sense, and included the following: i) gender, ii) age, iii) education level, iv) marital status, v) duration of stay in Thailand, vi) type of abode, vii) possession of a work permit, viii) possession of a legal ID (These include official passport, temporary registration card provided by Thai authorities. Myanmar National ID card is not included.) ix) occupation, x) Thai speaking ability, xi) knowing a place to test for HIV in one’s area, xii) knowing someone who died from AIDS, xiii) self-risk assessment of HIV, and xiv) HIV/AIDS knowledge. HIV/AIDS knowledge was assessed with 17 items on main routes of HIV transmission (e.g. can people protect themselves by using condoms? by abstaining from sexual intercourse? etc.), misconception about HIV/AIDS (e.g. can a person get HIV/AIDS through supernatural means? by sharing a meal with someone with HIV or AIDS? etc.), knowledge on HIV/AIDS disease progression (e.g. do you think that a healthy-looking person can also transmit HIV/AIDS?). A score was created by assigning the value 1 to correct answers and 0 to incorrect or don’t know answers. Scores equal to, or lower than the median are considered poor knowledge and scores higher than the median are regarded as good knowledge.

### Statistical Analysis

Statistical analyses were performed using SPSS for Windows 17.0 (SPSS Inc., Chicago, Illinoi, USA). The first part of the analysis focused on the sample of participants who were filtered to respond to the item “have you ever been tested for HIV?”. This question was exclusively asked to participants whose answers were “yes” to the filtering item “do you know any place proving counseling and testing for HIV?”. Univariate analysis was used to obtain descriptive statistics of the selected variables. Bivariate analyses using Chi-square or Fisher’s exact test for categorical variables and Mann Whitney U-test for continuous variables were performed to document the associations of covariates with the outcome of interest. In multivariate analysis we specified three regression models for HIV testing experience. The first model was a function of all variables that were significant in the bivariate analysis (*P* value < 0.05). The second model built upon the first model, and additionally included variables that were associated with HIV testing experience with *P* value ≤ 0.10. The third model was exhaustive and included all the variables. Adjusted odds ratios (AOR) of the covariates were provided along with their 95% confidence intervals (CI). The models were compared using the Akaike Information Criterion (AIC), which was manually computed using the formula. The optimal model among the candidates is the one with the smallest AIC score [39]. Fifteen cases were excluded from the analysis, of which 1 case had a missing value on the outcome variable, and 14 cases on key independent variables. There was no evidence of multicollinearity following the diagnostic procedures. The second part of the analysis was descriptive in nature and focused on participants whose answers were “don’t know” or “uncertain” to the filtering question. Additionally, we selected a number of items (restricted to MWs who reported having been tested for HIV) to assess the quality of the VCT (Voluntary Counseling and Testing) services in terms of the language used, the understanding of the counseling content in both the pre-and post-test counseling, and the confidentiality nature of the HIV test results.

## Results

### Participant characteristics

**Fig 1:**
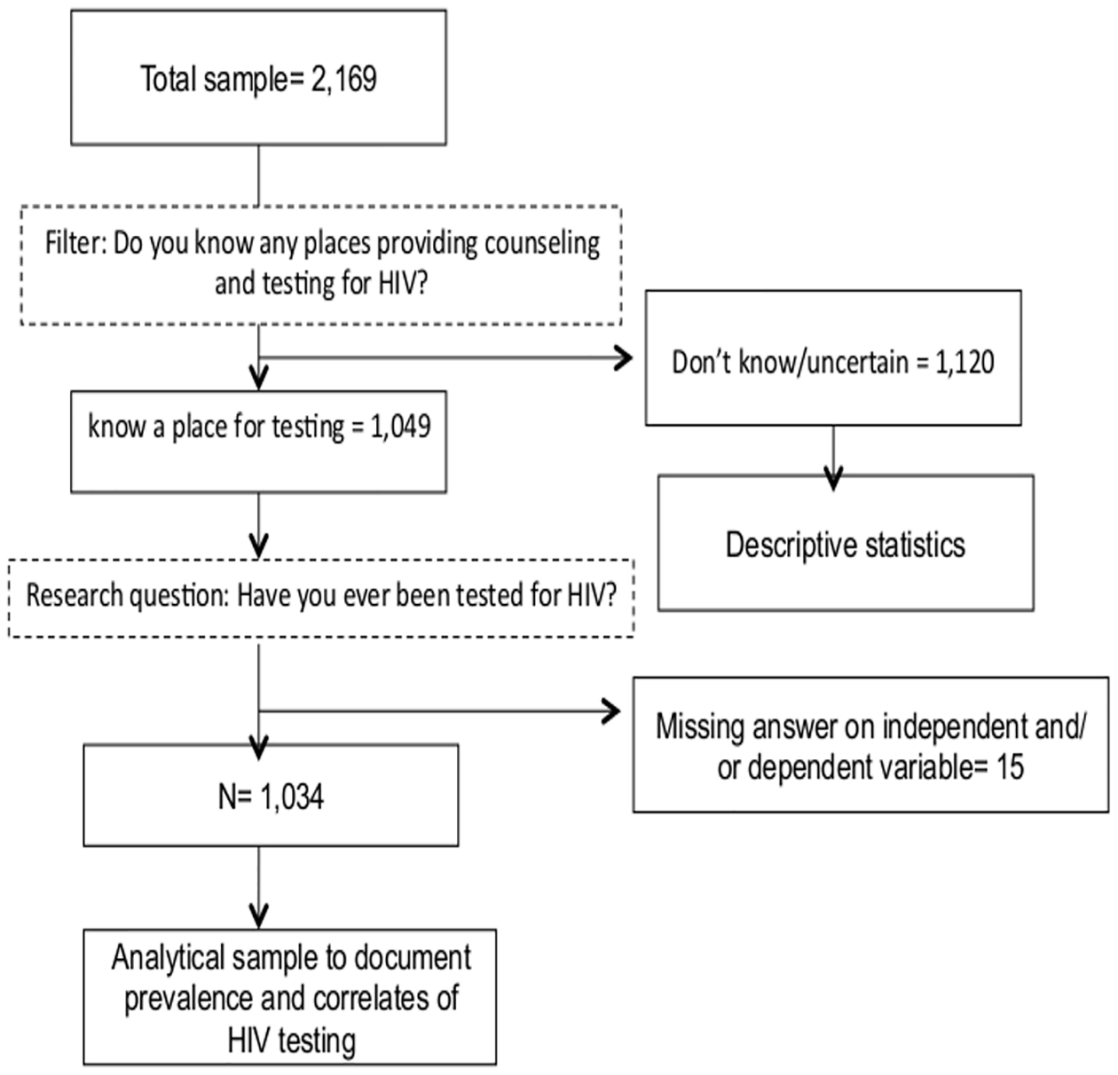
Flow of the data analysis.

Table 1 displays socio-demographic and behavioral characteristics of participants, and Fig 1 outlines the flow of the analysis. In total, 2,169 MWs from Myanmar completed the survey (the refusal rate was estimated to be less than 1%), of which 1,049 participants consisted of those who knew a place to test for HIV and 1,120 of those who did not or were not certain where to test for HIV. Of the participants who knew an HIV testing place, most had a primary or lower education level (73.8%), and over half lived with a partner (57.0%), and had resided in Thailand for less than 5 years (54.5%). The majority of participants had a work permit (85.3%) and a legal ID (92.9%), worked in factories (57.4%), lived outside of their working sites (62.1%), and were sexually active (74.4%). The median age in this group was 27 years [Interquartile range (IQR): 23–33], and there was a relatively higher proportion of males than females (58.8% vs 41.2%).

**Table 1:** Socio-demographic and behavioral characteristics of participants filtered by the item “do you know any places proving counseling and testing for HIV.”

	Knowing anyplace providing HIV test					
	Yes			No		
	Male	Female	Total	Male	Female	Total
	(N = 617)	(N = 432)	(N-1,049)	(N = 599)	(N = 521)	(N = 1,120)
**Education level**						
Primary school or lower	439 (71.2)	335 (77.5)	774 (73.8)	414 (69.1)	411 (78.9)	825 (73.7)
Secondary school or higher	150 (24.3)	69 (16.0)	219 (20.9)	101 (16.9)	68 (13.1)	169 (15.1)
Missing	28 (4.5)	28 (6.5)	56 (5.3)	84 (14.0)	42 (8.1)	126 (11.3)
**Marital status**						
Living with partner (married or not married)	290 (47.0)	308 (71.3)	598 (57.0)	241 (40.2)	243 (46.6)	484 (43.2)
Single	278 (45.1)	105 (24.3)	383 (36.5)	299 (49.9)	263 (50.5)	562 (50.2)
Married but living apart	31 (5.0)	4 (0.9)	35 (3.3)	52 (8.7)	6 (1.2)	58 (5.2)
Other	18 (2.9)	15 (3.5)	33 (3.1)	7 (1.2)	9 (1.7)	16 (1.4)
**Duration of stay in Thailand**						
0–59 months	341 (55.3)	231 (53.5)	572 (54.5)	402 (67.1)	376 (72.2)	778 (69.5)
≥ 60 months	276 (44.7)	201 (46.5)	477 (45.5)	197 (32.9)	145 (27.8)	342 (30.5)
**Type of abode**						
At the worksite	244 (39.5)	154 (35.6)	398 (37.9)	227 (46.2)	239 (45.9)	516 (46.1)
Outside worksite	373 (60.5)	278 (64.4)	651 (62.1)	322 (53.8)	281 (53.9)	603 (53.8)
Missing	0 (0.0)	0 (0.0)	0 (0.0)	0 (0.0)	1 (0.2)	1 (0.1)
**Work permit**						
No/Don’t know	80 (13.0)	74 (17.1)	154 (14.7)	116 (19.4)	149 (28.6)	265 (23.7)
Yes	537 (87.0)	358 (82.9)	895 (85.3)	483 (80.6)	372 (71.4)	855 (76.3)
**Legal ID**						
No/Don’t know	47 (7.6)	28 (6.5)	75 (7.1)	73 (12.2)	88 (16.9)	161 (14.4)
Yes	570 (92.4)	404 (93.5)	974 (92.9)	526 (87.8)	431 (82.7)	957 (85.4)
Missing	0 (0.0)	0 (0.0)	0 (0.0)	0 (0.0)	2 (0.4)	2 (0.2)
**Occupation**						
Factories	344 (55.8)	258 (59.7)	602 (57.4)	319 (53.3)	362 (69.5)	681 (60.8)
Agriculture	51 (8.3)	70 (16.2)	121 (11.5)	44 (7.3)	42 (8.1)	86 (7.7)
Fisheries/ Deep water seafarer	104 (16.9)	65 (15.0)	169 (16.1)	118 (19.7)	67 (12.9)	185 (16.5)
Others	118 (19.1)	39 (9.0)	157 (15.0)	118 (19.7)	50 (9.6)	168 (15.0)
**Ever had sex**						
Yes	452 (73.3)	328 (75.9)	780 (74.4)	340 (56.8)	260 (49.9)	600 (53.6)
No	165 (26.7)	104 (24.1)	269 (25.6)	259 (43.2)	261 (50.1)	520 (46.4)
**Has spouse/regular partner**^#^						
Yes	317 (70.8)	312 (95.1)	629 (81.1)	291 (86.1)	246 (95.7)	537 (90.3)
No	131 (29.2)	16 (4.9)	147 (18.9)	47 (13.9)	11 (4.3)	58 (9.7)
**Ever used condoms with spouse/ regular partner**^#^						
Yes	68 (21.5)	48 (15.4)	116 (18.4)	38 (13.1)	15 (6.1)	53 (9.9)
No	249 (78.5)	263 (84.3)	512 (81.4)	236 (81.1)	227 (92.3)	463 (86.2)
Missing	0 (0.0)	1 (0.3)	1 (0.2)	17 (5.8)	4 (1.6)	21 (3.9)
**Use of condom in the past 12 months in the last sex**^#^						
Yes	21 (61.8)	14 (58.3)	35 (60.3)	18 (81.8)	5 (100.0)	23 (85.2)
No	13 (38.2)	10 (41.7)	23 (39.7)	4 (18.2)	0 (0.0)	4 (14.8)
**Ever had sexual intercourse with non-regular partner in the past 12 months**^#^						
Yes	31 (6.9)	1 (0.3)	32 (4.1)	10 (2.9)	0 (0.0)	10 (1.7)
No	421 (93.1)	326 (99.4)	747 (95.8)	329 (96.8)	260 (100.0)	589 (98.2)
Missing	0 (0.0)	1 (0.3)	1 (0.1)	1 (0.3)	0 (0.0)	1 (0.2)
**Consistency of condom use with non-regular partner in the past 12 months**^#^						
Every time	11 (35.5)	0 (0.0)	11 (34.7)	2 (20.0)	0 (0.0)	2 (20.0)
Not every time	20 (64.5)	1 (100.0)	21 (65.6)	7 (70.0)	0 (0.0)	7 (70.0)
Missing	0 (0.0)	0 (0.0)	0 (0.0)	1 (10.0)	0 (0.0)	1 (10.0)
			Median (IQR)			Median (IQR)
Age (years)			27 (23–33)			25 (20–32)

^#^ Data restricted to the subgroup of sexually active participants

Of the 1,120 participants who did not know or were uncertain about a place to test for HIV, 825 (73.7%) had a primary education level or lower. The median age was 25 years (IQR: 20–32). Most participants were either single (50.2%) or lived with a partner (43.2%). The majority of participants in this group had resided in Thailand for less than 5 years (69.5%), had a work permit (76.3%), a legal ID (85.4%), worked in factories (60.8%), and had sexual experience (53.6%). Among the sexually active participants in this group, 537 (90.3%) had a spouse or regular partner, of whom the majority (86.2%) never used condoms with their spouse or regular partners. Compared to participants who knew a place for HIV testing, those who did not know (or were uncertain) appeared, to some extent, to have a lower risk of HIV/STI infection. The percentage of condom use in the past 12 months for those who last had sexual intercourse with a regular partner was higher (85.2% vs 60.3%) than that of participants who last had sexual intercourse with a non-regular partner. (1.7% vs 4.1%) Nevertheless, condom use with a regular partner (9.9%) and consistent condom use with a non-regular partner (20.0%) of this group of participants were fairly lower than those of participants who knew an HIV testing place (18.4% and 34.7%, respectively).

### Bivariate associations between independent variables and past HIV testing experience

The analysis to identify correlates of “ever been tested for HIV” included a total of 1,034 participants who knew a place to test for HIV. Fifty-five participants (5.3%) reported having been tested for HIV. MWs with a secondary or higher education level [odds ratio (OR), 1.90; CI, 1.06–3.42; *P* = 0.028)], those who had resided in Thailand for five years or more (OR, 2.80; CI, 1.56–5.04; *P* = 0.000), lived outside of the work site (OR, 2.04; CI, 1.08–3.85; *P* = 0.025), and who were able to speak Thai (OR, 2.92; CI, 1.41–6.03; *P* = 0.003), were more likely to have been tested for HIV. Participants who were single were less likely to ever have been tested for HIV (OR, 0.45; CI, 0.23–0.873; *P* = 0.016) compared to those who lived with partner. MWs who worked in the fishery sector were more than two times likely to report ever having been tested for HIV compared to those who worked in factories (OR, 2.51; CI, 1.35–4.67; *P* = 0.003). (Table 2)

**Table 2:** Bivariate associations socio-demographic and psychosocial variables with ever have been tested for HIV.

	Ever have been tested for HIV				P value^a^
	Yes (n = 55)	No (n = 979)	Total (n = 1,034)	Crude OR (95% CI)	
	n (%)	n (%)	n (%)		
**Sex**					
Male	26 (47.3)	580 (59.2)	606 (58.6)	1.00	
Female	29 (52.7)	399 (40.8)	428 (41.4)	1.621 (0.94–2.79)	0.079
**Educational level**					
Primary school or less	37 (67.3)	780 (79.7)	817 (79.0)	1.00	
Secondary school or higher	18 (32.7)	199 (20.3)	217 (21.0)	1.90 (1.06–3.42)	0.028
**Marital status**					
Living with partner (married or not)	40 (72.7)	550 (56.2)	590 (57.1)	1.00	
Single	12 (21.8)	365 (37.3)	377 (36.5)	0.45 (0.23–0.873)	0.016
Married living apart	1 (1.8)	33 (3.4)	34 (3.3)	0.41 (0.05–3.126)	0.718^b^
Other	2 (3.6)	31 (3.2)	33 (3.2)	0.88 (0.20–3.84)	1.000^b^
**Duration of stay in Thailand**					
0–59 months	17 (30.9)	545 (55.7)	562 (54.4)	1.00	
≥ 60 months	38 (69.1)	434 (44.3)	472 (45.6)	2.80 (1.56–5.04)	0.000
**Type of abode**					
At the worksite	13 (23.6)	379 (38.7)	392 (37.9)	1.00	
Outside worksite	42 (76.4)	600 (61.3)	642 (62.1)	2.04 (1.08–3.85)	0.025
**Work permit**					
Yes	47 (85.5)	835 (85.3)	882 (85.3)	1.00	
No/Don’t know	8 (14.5)	144 (14.7)	152 (14.7)	0.98 (0.45–2.13)	0.973
**Legal ID**					
No/Don’t know	2 (3.6)	70 (7.2)	72 (7.0)	1.00	
Yes	53 (96.4)	909 (92.8)	962 (93.0)	2.04 (0.48–8.55)	0.423^b^
**Occupation**					
Factory	28 (50.9)	567 (57.9)	595 (57.5)	1.00	
Agriculture	1 (1.8)	119 (12.2)	120 (11.6)	0.17 (0.02–1.26)	0.070^b^
Fisheries/deep water seafarer	18 (32.7)	145 (14.8)	163 (15.8)	2.51 (1.35–4.67)	0.003
Others	8 (14.5)	148 (15.1)	156 (15.1)	1.09 (0.48–2.45)	0.826
**Thai speaking ability**					
No	9 (16.4)	356 (36.4)	365 (35.3)	1.00	
Yes	46 (83.6)	623 (63.6)	669 (64.7)	2.92 (1.41–6.03)	0.003
**HIV/AIDS knowledge**					
≤ 13	23 (41.8)	535 (54.6)	558 (54.0)	1.00	
> 13	32 (58.2)	444 (45.4)	476 (46.0)	1.67 (0.96–2.90)	0.063
**Know someone who died of AIDS**					
No/Don’t know	27 (49.1)	681 (69.6)	708 (68.5)	1.00	
Yes	28 (50.9)	298 (30.4)	326 (31.5)	2.37 (1.37–4.09)	0.001
**Self-risk assessment**					
No risk	46 (83.6)	888 (90.7)	934 (90.3)	1.00	
Other^§^	9 (16.4)	91 (9.3)	100 (9.7)	1.90 (0.90–4.02)	0.084
**Ever had sex**					
No	21 (38.2)	351 (35.9)	372 (36.0)	1.00	
Yes	34 (61.8)	628 (64.1)	662 (64.0)	0.90 (0.51–1.58)	0.726
	Median (IQR)	Median (IQR)	Median (IQR)		
**Age (years)**	29 (25–33)	27 (23–33)	27 (23–33)		0.087^c^

OR, odds ratio; CI, confidence interval; ID, identity;

^a^ *P* values based on chi-square test of proportions unless otherwise specified;

^b^ Fisher exact test;

^c^ *P* values based on the Mann Whitney U-test;

^§^ Other includes “a lot risk” and “a little risk”

Table 3 displays reasons for testing for HIV by gender. Most MWs (75.9%) cited pregnancy as the reason for testing, followed by curiosity (13.8%), and as part of the job application process (10.3%). Regarding male migrant participants, curiosity (46.4%), blood donation (25.0%), and job application process (21.4%) were the most frequently cited reasons for testing.

**Table 3:** Reasons for testing for HIV.

	Frequency		
Reason	Male (N = 28)	Female (N = 29)	Total (N = 57)
	n (%)	n (%)	n (%)
Pregnant	N/A	22 (75.9)	22 (38.6)
Work application	6 (21.4)	3 (10.3)	9 (15.8)
Marriage	1 (3.6)	0 (0)	1 (1.8)
Partner has risk behavior	1 (3.6)	0 (0)	1 (1.8)
I have risk behavior	1 (3.6)	0 (0)	1 (1.8)
Part of routine health exam	1 (3.6)	0 (0)	1 (1.8)
Surgery/ill	1 (3.6)	0 (0)	1 (1.8)
Blood donation	7 (25.0)	0 (0)	7 (12.3)
Curiosity	13 (46.4)	4 (13.8)	17 (29.8)
Coerced			
No reason			
Other	3 (10.7)	0 (0)	3 (5.3)

Multiple responses are possible; N/A: not applicable

### Multivariate analysis

In the multivariable analysis (Model 3), having a secondary or higher education level (AOR, 2.58; CI, 1.36–4.90; *P* = 0.004), having resided in Thailand for at least five years (AOR, 2.45; CI, 1.25–4.78; *P* = 0.009), knowing someone who died of AIDS (AOR, 1.81; CI, 1.00–3.27; *P* = 0.048), and working in the fishery sector (AOR, 2.51; CI, 1.28–4.92, *P* = 0.007) remained significant. Being female (AOR, 1.96; CI, 1.05–3.66; *P* = 0.033) and not having a work permit (AOR, 3.71; CI, 1.36–10.13; *P* = 0.010) were associated with increased odds of ever having been tested for HIV. Additionally, there was a trend for participants who resided outside the work site to more likely have been tested for HIV compared to those who lived in the work site (AOR, 1.82; CI, 0.93–3.59; *P* = 0.079). (Table 4)

**Table 4:** Multivariate analysis of factors associated with ever have been tested for HIV.

	Models		
	Adjusted OR (95%CI)		
	(1)	(2)	(3)
**Educational level**			
Primary school or less	1.00	1.00	1.00
Secondary school or higher	2.49 (1.33–4.65)*	2.58 (1.37–4.86)*	2.58 (1.36–4.90)*
**Marital status**			
Living with partner (married or not)	1.00	1.00	1.00
Single	0.57 (0.28–1.14)	0.63 (0.29–1.38)	0.61 (0.27–1.34)
Married living apart	0.55 (0.71–4.31)	0.70 (0.87–5.65)	0.63 (0.07–5.20)
Other	0.98 (0.21–4.41)	1.15 (0.25–5.36)	1.00 (0.21–4.74)
**Duration of stay in Thailand**			
0–59 months	1.00	1.00	1.00
≥ 60 months	2.28 (1.19–4.33)*	2.50 (1.28–4.88)*	2.45 (1.25–4.78)*
**Type of abode outside worksite** (vs at worksite)	1.76 (0.91–3.39)^†^	1.72 (0.88–3.34)	1.82 (0.93–3.59)^†^
**Thai speaking ability** yes (vs no)	1.59 (0.74–3.44)	1.73 (0.79–3.79)	1.88 (0.85–4.17)
**Know someone who died from AIDS** yes (vs no/don’t know)	1.67 (0.95–2.96)^†^	1.70 (0.95–3.06)^†^	1.81 (1.00–3.27)*
**Occupation**			
Factory	1.00	1.00	1.00
Agriculture	0.22 (0.29–1.67)	0.22 (0.29–1.71)	0.11 (0.01–1.00)^†^
Fisheries/Deep water seafarer	2.32 (1.22–4.41)*	2.61 (1.35–5.04)*	2.51 (1.28–4.92)*
Other	1.13 (0.49–2.58)	1.32 (0.56–3.09)	1.20 (0.50–2.85)
**Self-risk assessment**			
No risk		1.00	1.00
Other^§^		1.33 (0.60–3.2.95)	1.47 (0.66–3.30)
**Age (increase by one year)**		0.98 (0.93–1.02)	0.98 (0.94–1.03)
**Sex**			
Male		1.00	1.00
Female		1.95 (1.05–3.59)*	1.96 (1.05–3.66)*
**HIV/AIDS knowledge**			
≤ 13		1.00	1.00
> 13		1.59 (0.89–2.84)^†^	1.54 (0.86–2.78)
**Work permit**			
Yes			1.00
No/don’t know			3.71 (1.36–10.13)*
**Legal ID**			
No/don’t know			1.00
Yes			2.22 (0.40–12.21)
**Ever had sex**			
No			1.00
Yes			0.79 (0.43–1.44)
AIC score	397.622	397.252	397.144

Adjusted OR, Adjusted odds ratio; CI, confidence interval; ID, identity; AIC, Akaike Information Criterion;

* *P* value < 0.05;

^†^*P* value <0.10>0.05;

^§^ includes those who were at risk and those whose answers were “uncertain”, and nonresponse.

### Quality of VCT services

The sample included 26 participants who tested for HIV in the last 12 months. Fifteen (57.7%) participants and 16 (61.5%) participants respectively reported having received pre-test and post-test counseling. The majority of counseling was conducted in Burmese, and most participants reported that they understood the content of both pre-test (93.3%) and post-test (93.7%) counseling. Twenty-three (88.5%) participants received the result of the test, of which 14 (60.9%), reported that the result was provided in the presence of a third party (e.g. partner/family, friend/co-worker, etc) (S1 Table)

## Discussion and Implications

There is a remarkable dearth of studies addressing HIV testing among cross-border MWs in Thailand, and in Southeast Asia at large. The current study leveraged data from the PHAMIT-2 Project to document the prevalence of, and factors associated with, HIV testing among MWs from Myanmar.

In this study, the odds of ever having been tested for HIV was higher for participants who had at least a secondary education level. Our finding corroborates results from a previous study in Kenya in which individuals with secondary and higher education attainment were more likely to have been tested for HIV than those with no or lower education levels [40]. There is a general trend for individuals with higher education levels to use more health care services [41–43]. In other settings, higher education levels were associated with higher knowledge on HIV/AIDS prevention, transmission and HIV testing [23, 44], and the latter is documented as a predictor for HIV testing [45, 46]. However, in our study, there was no association between knowledge on HIV/AIDS and HIV testing. Nonetheless, interventions that aim to improve HIV/AIDS-related knowledge and awareness on the benefits of HIV testing are an important component of HIV prevention, and should be delivered in a way that is accessible to those with little or no formal education.

We also found that females were almost two times more likely to have been tested for HIV compared with males. This is in line with findings from other studies showing gender differentials in HIV testing behavior, and fits well with the common observation that females have more contact with health care systems, particularly because they attend routine antenatal care as part of the sexual and reproductive health services [47–49]. Additional evidence in support of gender differentials in HIV testing in our study includes the fact that female MWs overwhelmingly cited pregnancy as the reason for testing, contrasting with male MWs who mostly tested for HIV out of curiosity, for blood donation, or as a part of the work application process. The uptake of HIV services by men has proven to be a challenge in many settings, and there exists evidence showing that migrant men are less exposed to HIV testing and less willing to be tested [50]. Our study suggests the need to consider gender differences in the design of intervention programs to improve the uptake of HIV testing services among MWs. Interventions such as those that strengthen antenatal care services as an entry point for HIV testing can potentially benefit both females and males if they are delivered in a framework that targets couples for testing. Interventions should also target female and male MWs outside the context of antenatal care and/or sexual reproductive health services. For example, mobile HIV testing outlets organized in the vicinity of, or at worksites could be a promising strategy to boost HIV testing in male MWs.

In our study, MWs without a work permit had higher odds of ever having been tested for HIV compared to those who possessed a work permit. It is not clear why this was the case; however, considering the characteristics of participants presented in Table 1, 265 of 419 participants (63.2%) without a work permit reported that they did not know any place providing counseling and testing for HIV. This proportion was fairly higher than that of participants who possessed a work permit, 855 of 1,750 participants (48.8%), implying that “knowing a place for HIV testing” was still an important barrier of accessing HIV testing for MWs without a work permit than those with a work permit. Thus, we might have selected in the group of participants without a work permit, those who were more likely to have been tested for HIV because our analysis was restricted to the group of participants who knew a place for HIV testing. There is however still need for future research to explain the observed difference in HIV testing between MWs with and without a work permit in Thailand. In contrast, MWs with legal ID were more likely to have been tested for HIV; however, the association was not statistically significant. In other settings, legal factors such as the lack of entitlement to health service for undocumented factors, or the fear that disclosure about HIV may adversely affect legal status were documented as important factors that increased the vulnerability to HIV infection among the population [51].

Knowing someone who is infected with, or who died of HIV/AIDS may influence individuals’ perceived risk of HIV risk, which in turn, depending on studies, was shown have been associated with increased or decreased odds of testing for HIV [52–56]. These variables have been meaningfully used as an indicator to help contextualize individual HIV risk perception in many settings [57–59]. In our study, participants who knew someone who died of AIDS had increased odds of ever having been tested for HIV. A previous study showed that, across many African countries, participants who reported that they did not know someone who has AIDS or died of AIDS were much less likely to report ever having been tested and to receive their HIV test results [60]. However, HIV risk perception was not associated with ever having been tested for HIV in our study. This is an indication that factors other than risk perception may mediate the association of knowing someone who died of AIDS with ever having been tested for HIV. Moreover, there is a possibility of residual confounding due to insufficient sensitivity of our measure of HIV risk perception. Furthermore, although there is a well-established relationship between self-perception of HIV risk and uptake of safer behaviors [60], this association suffers from a lot of inconsistencies [61–63]. Nevertheless, interventions designed to increase risk perception, more specifically HIV risk personalization, should be one of the prongs of strategies to promote HIV testing among MWs.

In this study, working in the fishery sector (fisheries/deep water seafarer) was independently associated with increased odds of ever having been tested for HIV. Migrant fishermen in particular have been identified as a group at high risk of HIV infection among the MWs in Thailand. For example, seafarers are more likely to visit sex workers than other migrant groups and to report lower use of condoms particularly when acquainting with non-regular partners [21, 64]. In a qualitative study, migrant seafarers reported that many of them went for testing because they felt susceptible to HIV; however, many others were reluctant to testing due to fear of a positive result or other inconveniences such as time and money [22]. The same study indicated that seafarers were aware of their risk of HIV infection especially from sex workers, and most had observed their peers become sick and die from AIDS. This could to some extent explain our finding that knowing someone who died of AIDS was associated with increased odds of ever having been tested for HIV.

Although not statistically significant, there was a trend for MWs residing outside of the working site to more likely report ever having been tested for HIV compared to those who lived in the work site. This finding is of particular relevance as it emphases the need for interventions to reach out to MWs who work and live at their workplace, and as a consequence, have limited exposure to health programs that are routinely provided to the community. As outlined earlier, provision of mobile HIV testing and counseling to MWs at their workplace can possibly increase the proportion of HIV testing in this population. Although not related to MWs, results from a community-based intervention strongly demonstrated the efficacy of mobile voluntary HIV counseling and testing (MVCT) provided free of charge and associated with community mobilization to increase the HIV testing rate in Northern Thailand rural communities [65]. In the contexts of the PHAMIT-2 project, with collaborations between NGOs, the local Public Health Office and the hospital located in the area, MVCT also operated with a focus on migrants living in the community and in the workplace. The latter group was, however, more difficult to access for the project, in most cases due to non-cooperation or disapproval from the employer.

Previous research has linked language barriers to limited access to health services, including uptake of HIV-testing service [49]. Poor or limited ability of the language in the host country poses a serious challenge for MWs to accessing health care services. In our study, however, Thai speaking ability was not associated with HIV testing in the multivariate models. It is important to note that although the majority of participants who were tested for HIV in the last 12 months, and received pre-and post-test counseling, reported that counseling was offered in the Burmese language, and that most understood the content of both pre-and post-counseling sessions, a substantial proportion of MWs did not receive pre-and post-test counseling. This suggests that language barrier or unavailability of the counseling in the MWs’ native language is still a possible reason to explore further, and that provision of migrant-friendly services, such as those delivered in the migrant’s native language, remains important (See S1 Table). In addition to providing language-sensitive HIV testing services, the quality of HIV testing services will greatly increase by ensuring confidentiality of the test results. In our study, the majority of test results were not provided in strict confidentiality (S1 Table).

Overall, we found that a low proportion of MWs from Myanmar had previously tested for HIV. Other studies in Asia have reported a low proportion of HIV testing among the migrant population. For example, in China, only one out of 100 male migrants had previously been tested [13, 66]. In our study, the actual proportion of those who had ever been tested could even be lower than that reported under the provision that those who did not know any place providing HIV test are considered as never having been tested for HIV. The low proportion of HIV testing in MWs from Myanmar in this study is a strong call for strategies to strengthen HIV testing and counseling services for the MWs in Thailand in general. Interventions should be tailored to the specific needs of MWs, and should be grounded in outreach and community mobilization that integrate social, cultural, and structural contextual factors that shape the health-seeking behavior of MWs in Thailand. In the PHAMIT-2 Project, one of the key goals is to increase the HIV testing rate of MWs in Thailand through a range of multi-level and multi-stakeholder activities. For instance, the PHAMIT-2 Project intends to increase the uptake of VCT services through field staff and peer outreach who refer MWs to VCT services at drop-in centers or community hospitals, strengthened by mobile VCT services operated by hospitals or health center staff. These activities are synergistically linked with other core activities of the Project, which indirectly have an impact on HIV testing behavior [37].

There are a number of limitations worth mentioning. Firstly, our study is cross-sectional by nature; thus, causation cannot be inferred. Secondly, our outcome measure "ever been tested for HIV", while being useful indicator to assess the overall reach of HIV testing services, does not capture recent trends in HIV test-seeking behavior as would a time-bound indicator like "tested in the last 12 months". Thirdly, participants were not randomly selected and are, therefore, unlikely to be representative of all MWs from Myanmar although those included may have been more likely to have been tested given the manner of their selection. Fourthly, because of the small proportion of participants who reported ever having been tested for HIV in our sample, we may not have sufficient statistical power to assess the relationship between the predictors and our outcome of interest. Moreover, the survey questionnaire was designed in such a way that participants who were asked if they had ever been tested for HIV were filtered from those who knew a place to test for HIV. Consequently, the analysis to identify correlates of "ever been tested for HIV" was conducted in only around half of the participants that were recruited. We recognize that this could be a source of significant bias; however, the degree to which our results may have been affected could not be determined. When assuming that participants who did not know any place providing HIV testing had never been tested for HIV, the proportion of those who reported "ever been tested for HIV" was substantially reduced (2.5%), and we could not perform the multivariate analysis. Fifthly, Of the 2,169 Myanmar respondents, 42.6% were Burmese, 22.9% were Dawei or Tavoya, 21.6% were Mon, 9.4% were Karen, 1.8% were Pa-oh, 1.3% were Ya-kai, and 0.3% were Shan. The fact the interviews were conducted in Burmese, at the exception of the Mon who were interviewed in Mon language, could be a possible source of selection bias given that those with no or limited ability to speak Burmese might have been excluded from the study. Additionally, the variable “ethnicity”, although epidemiologically important for our study, was not included in our analysis given that some ethnic groups who had no or too few outcomes, responded yes on the HIV testing item. There is a possibility that social desirability and recall bias might have affected our results given that the collected data were self-reported. Lastly, issues such as “health insurance status”, “discrimination”, “stigma”, and “security”, which are important for the uptake of HIV testing, were not addressed in the survey; therefore, could not be controlled in our models. Further research is needed to complement our findings by addressing the above limitations.

## Conclusion

The main finding from our study is that a significantly low proportion of Thailand’s MW population from Myanmar has ever been tested for HIV. This sounds the alarm for the need to rapidly develop strategies to improve the up-take of HIV testing services in this population, and thus secure the benefits associated with awareness of one's HIV status. We have identified a number of individual-level factors (such as gender, education, and lack of knowledge about HIV testing sites) and structural barriers (limited availability of HIV test counseling in migrants’ native language) that can inform future interventions to improve HIV testing among MWs in Thailand. Such interventions should include culturally-sensitive HIV/AIDS and health education that encourages safe sexual practices, facilitates the flow of information about places for HIV testing sites, and ensures availability of language assistance and confidentiality of HIV testing.

## Supporting Information

S1 Table Quality of Voluntary Counseling and Testing. (DOCX)
