# Supplementary material for: Correlates of HIV Testing Experience among Migrant Workers from Myanmar Residing in Thailand: A Secondary Data Analysis
Source: PLoS One. 2016 May 3;11(5):e0154669. doi: 10.1371/journal.pone.0154669 (PMC4854405; doi:10.1371/journal.pone.0154669)
Supplement: S1 Table — (DOCX) [file pone.0154669.s001.docx]

**S1 Table. Quality of Voluntary Counseling and Testing (VCT)**

|  | Frequency (N=26) n (%) |
| --- | --- |
| **Pre-test counseling** |  |
| Yes | 15 (57.7) |
| No | 11 (42.3) |
| **Pre-test counseling language** |  |
| Burmese | 11 (73.3) |
| Thai | 4 (26.7) |
| **Understanding of the pre-test counseling** |  |
| Yes | 14 (93.3) |
| No | 1 (6.7) |
| **Post-test counseling** |  |
| Yes | 16 (61.5) |
| No | 10 (38.5) |
| **Post-test counseling language** |  |
| Burmese | 12 (75.0) |
| Thai | 4 (25.0) |
| **Understanding of the post-test counseling** |  |
| Yes | 15 (93.7) |
| No | 1 (6.3) |
| **Reception of the test result** |  |
| Yes | 23 (88.5) |
| No | 3 (11.5) |
| **Confidentiality at reception of the results** |  |
| Yes | 9 (39.1) |
| No | 14 (60.9) |

This table includes participants who tested for HIV in the last 12 months (N=26)
